# Self- and surrogate-seeking of information about mental health and illness in Germany

**DOI:** 10.1186/s12889-023-14998-0

**Published:** 2023-01-10

**Authors:** Anna Freytag, Eva Baumann, Matthias Angermeyer, Georg Schomerus

**Affiliations:** 1 Department of Journalism and Communication Research, Hanover University of Music, Drama and Media, Expo Plaza 12, 30539 Hannover, Germany; 2 Center for Public Mental Health, Untere Zeile 13, 3482 Gösing am Wagram, Austria; 3 Department of Psychiatry and Psychotherapy, University of Leipzig, Semmelweisstraße 10, 04103 Leipzig, Germany

**Keywords:** Mental health, Information-seeking, Information behavior, Surrogate-seeking, Logistic regression

## Abstract

**Background:**

Seeking information on mental health issues – both for oneself and on behalf of others (so-called surrogate-seeking) – is a critical early step in dealing with mental illness and known to impede stigmatizing attitudes and foster help-seeking. Yet, knowledge about mental health tends to be insufficient worldwide. Therefore, it is necessary to better understand the search for mental health information and examine the factors that are positively associated with information-seeking.

**Method:**

In a face-to-face survey in Germany (*N* = 1,522), we investigated the factors related to mental health information-seeking. The data was analyzed by means of a logistic regression model, in which we distinguished those searching information for themselves from so-called surrogate seekers, i.e., people who seek information on behalf of someone else.

**Results:**

Twenty-six percent of German adults in our sample have already searched for information on mental health, with the majority already having searched for information for others (73% of all seekers). Our findings indicate that individuals’ proximity to people with mental health issues, including their own mental health treatment experience (Cramer’s *V* = .429, *p* < .001), education (Cramer’s *V* = .184, *p* < .001), and desire for social distance from the affected people (*F* [1, 1516] = 73.580, *p* < .001, η^2^ = .046), play an important role in mental health information-seeking. The patterns of sociodemographic and proximity factors hereby differ between self-seekers and surrogate-seekers.

**Conclusions:**

Our study provides insights into the public’s mental health information orientation. The findings may particularly guide strategies to improve mental health awareness and fill knowledge gaps in supporting informed decision-making and reducing stigma. Surrogate seekers appear to be an important and distinctive target group for mental health information provision. Depending on whether one wants to promote surrogate- or self-seeking seekers, different target groups and determinants should be addressed.

**Supplementary Information:**

The online version contains supplementary material available at 10.1186/s12889-023-14998-0.

## Background

Acquisition of mental-health-related knowledge is a critical early step in dealing with mental illness. Seeking information on mental health issues – both for oneself and on behalf of others (so-called surrogate-seeking; [1]) – is known to impede stigmatizing attitudes and foster help-seeking [2–5]. Thus, there is a mutually reinforcing and dynamic interplay between mental health information-seeking, mental health stigma, and help-seeking [6–8]. However, studies reveal poor public knowledge about mental disorders and help options [9, 10]. While barriers to help-seeking have been examined in some detail [6, 11, 12], research is far less with respect to (surrogate) information-seeking for mental health problems.

Building on theoretical models of health information and surrogate-seeking in general as well as on previous empirical studies in the field (e.g., [3, 13–15]), the present study aims to examine which factors are associated with self and surrogate mental health information-seeking. In the following paper, we will review the state of theory and research on self and surrogate mental health information-seeking and derive a set of potentially relevant sociodemographic and proximity factors influencing both behaviors. We will then present our empirical study that examines the determinants of (surrogate) mental health information-seeking in a representative German population sample. The present manuscript contributes to both theory-building regarding the explanation of mental health information-seeking and, additionally, data that should help develop targeting strategies to foster awareness and stigma reduction, knowledge gain, and informed decision-making in the context of mental health and illnesses.

### Explaining mental health information-seeking

In this paper, *health information-seeking* is defined as “[…] active efforts to obtain specific information in response to a relevant event” [16]. It is thus understood as a purposeful and goal-oriented activity [16, 17] that, among others, helps to cope with health-related uncertainties, find strategies for living with health threats, and make self-determined, informed health-related decisions [18, 19].

The term health information is very broad and encompasses a variety of specific types of information. Health information can relate to health and/or illness in general or to specific diseases, medical procedures, treatments, prevention possibilities, public health, and topics such as unhealthy and healthy lifestyles [20]. In regard to *mental health, information-seeking* covers topics such as different mental illnesses, therapies, and the search for psychiatrists, clinics, or support resources in general but also information about preventative measures to maintain mental health. Numbers on how many people seek mental health information are scarce and mixed, and they mainly refer to online health information-seeking. According to Powell and Clark [15], only 11% of adults in the UK have ever used the Internet for searching mental health information. Scherr and Goering [21] noted that mental health information-seeking is even lower among users of a mood-tracking app. However, according to a study by the Pew Research Center, the percentage of US citizens searching the Internet for information on depression, anxiety, stress, or other mental health issues has increased significantly, from 12% in 2002 to 21% in 2009 [22].

Mental health information is relevant for both affected and non-affected persons. On one hand, mental health information can help affected people deal with their disease and make decisions regarding their care [23, 24]. Prior research shows that the desire for information regarding their mental illness and treatment as well as for taking part in the decision-making process is especially high among young patients [25]. Overall, seeking mental health information can be considered an important first step in the help-seeking process [3, 21]. Before a diagnosis, information-seeking can help affected individuals find out how severe their current symptoms are and what subsequent steps are possible [26]. Further, as we know from other diseases, information-seeking can help affected persons find support services and therapy options and assure therapy adherence in the long term [27, 28]. According to Cunningham and colleagues [29], people who are better informed about mental health problems are more likely to use services. Yet, information seeking is not strictly limited to people wanting to initiate services or therapy. It is an action taken during the whole episode of illness [30], and hereby by both treatment initiators, treatment repeaters (i.e., those using services once again), and people who do not use mental health treatment services, (i.e., those trying to find alternative ways to deal with their symptoms) [24, 29].

Additionally, mental health information can also be important for unaffected individuals. Several studies have revealed that knowledge of mental health is associated with more positive attitudes toward those affected by mental illness, with lower levels of mental health stigma and a higher willingness to seek professional mental health services [2, 31, 32]. However, past research has repeatedly exposed a deficiency in people’s mental health knowledge and literacy, manifesting in the non-recognition of disease symptoms, misconceptions about mental illnesses, and a lack of knowledge about the appropriate measures to help [9, 10, 33–38].

In summary, the search for mental health information has a positive impact on both affected and unaffected individuals and plays a crucial role in empowering (future) patients. Therefore, it is necessary to better understand the search for mental health information and examine the factors that encourage and promote information-seeking. Most theoretical models (e.g., the Comprehensive Model of Information Seeking [39], the Risk Information Seeking and Processing Model [40]; the Theory of Motivated Information Management [41], or the Planned Risk Information Seeking Model [42]) aiming to explain health information-seeking behaviors include an individual’s predisposing characteristics that may lead to engagement in health information-seeking [43]. Thereby, sociodemographic and socioeconomic characteristics, as well as health-related and cognitive factors, are taken into account. Most studies show that women, younger people, and people with higher education are more likely to search for health information in Europe [13, 19, 44–47], the United States [48–50], and Asian countries [51–53]. In addition to these personal factors, individual-level health-related and cognitive factors [54] are known to impact information-seeking. These include, for example, health status [19, 55] or prior use of health care services [56]. However, it must be noted that previous studies on the influence of health status on health information-seeking have yielded mixed results. Some studies report that individuals with poorer health status or chronic disease are less likely to seek health information [44, 57], while others showed increased motivation to seek information when affected or in treatment [13, 19, 55].

Due to the complexity and stigma surrounding mental health-related issues, it can be assumed that patients are likely to investigate and search for support regarding their problems online [58]. Thus, most studies on determinants of mental health information-seeking focus on the Internet as an information source. For instance, Chisolm [59] found that male gender and age over 65 are negatively associated with searching for mental health-related information on the Internet. Eichenberg and colleagues [14] support these findings: Their study revealed that young and single persons with a final degree and with an above-average income were most likely to turn to the Internet in case of mental health-related needs. Lannin and colleagues [60], moreover, showed that adolescents who identified as Black/African Americans were half as likely to seek mental health information online as adolescents identifying with another race/ethnicity.

In addition to sociodemographic determinants, both Lannin et al. [60], Chisolm [59], as well as Khann and colleagues [61] noted that distress among adolescents and adults, such as anxiety or functional impairment, was a statistically significant predictor of the decision to seek online mental health information. The role of mental health distress was also emphasized by Powell and Clarke [15], who found that “internet users with current experience of mental health distress were more likely to have used the Internet to find information about a mental health issue than those without current mental health distress” [15]. Other studies correspond to these results and demonstrate that affliction with a mental illness, or at least the occurrence of symptoms of a mental illness, increases the propensity of searching online for mental health-related information [21].

Previous research has also investigated the inhibiting factors of mental health information-seeking. Lannin and colleagues [3] identified “self-stigma as an important barrier to initial decisions to seek information about mental health concerns and counseling, even for individuals with higher levels of distress” [3]. However, stigma did not consistently and significantly predict individuals’ decisions to seek mental health information online [60]. In addition to general attitudes toward seeking mental health information, self-stigma may also affect the type of information sought, as, for instance, information about online support groups has a different stigmatizing effect on the self than information about specialized care services.

To conclude, previous research shows that sociodemographic, health- and stigma-related characteristics determine mental health information-seeking. Especially age, gender, education, and relationship status seem to represent sociodemographic factors, while an individual’s level of mental distress and stigmatizing attitudes appear to be relevant determinants at the cognitive level. Other forms of proximity to the topic of mental health, such as affected persons in the immediate environment or professional contacts, have not yet been examined in prior research but, based on the findings to date, also seem worth investigating. Therefore, in the context of this study, the following first research question will be pursued:

RQ1: How do non-seekers of mental health information differ from mental health information seekers regarding (a) sociodemographics (age, gender, education, relationship status, and migration background) and (b) their proximity to the topic of mental health and illness (personal mental health status, professional contacts, people affected in social environment, and social distance)?

### Surrogate information-seeking

As an important prerequisite for an informed patient and as a known benefit for help-seeking, adherence to therapy, and more appropriate use of health care services, searching for mental health information is crucial for individuals who (might) suffer from mental health burdens or illness. However, not all people are equally motivated or have the same abilities or opportunities (e.g., because of their [mental] health situation) to obtain information independently on the Internet or from other sources of health information [44]. Besides health professionals and the media, also family members and friends support and significantly contribute to the patient’s (mental) health information status; they provide additional information or filter the relevant material from the flood of available information and evaluate it if the related person is unable or nonmotivated to do so, e.g., due to a lack of health and media literacy or knowledge of current health status [62]. As part of the social support system (see network-episode model, [30]), such surrogate seekers play a crucial role regarding access to help-seeking, the healing and therapy process of patients, and prevention measures.

It has been shown that people mostly appreciate this type of support [63, 64] and that informed family members positively impact patients’ health status and behaviors [65]. Studies on the prevalence of surrogate-seeking showed that in both the US and Europe, about 40 percent to 60 percent of people who search for health information on the Internet do so not only for themselves but also for others [1, 66, 67]. Those studies further suggest that certain demographic characteristics, such as gender and education of the person who seeks the information are relevant influencing variables: Women and higher educated individuals are more likely to be among the so-called surrogate seekers [62, 66, 68]. However, since other studies did not find these effects (e.g., 1, 67), evidence on surrogate health information-seeking is still somewhat ambivalent and inconsistent.

Besides demographic characteristics, surrogate-seeking for health information is shaped not only by individual characteristics of the seekers but also by relational factors, such as closeness to the patient or the person on behalf of whom the search is made [67, 69]. Concerning mental health, relationship closeness could also be understood more broadly as persons’ proximity to the topic as a whole, that is, for example, the extent to which they themselves are affected and have affected persons in their private environment, being professionally or voluntarily involved in the care or treatment of people with mental illness (i.e., professional contact), emotional closeness or distance, or even defensive or stigmatizing attitudes toward people affected from mental illnesses.

To the best of our knowledge, the field of mental health information has no studies on surrogate-seeking yet, and thus no data on how widespread this form of information behavior is and what individual factors are associated with it. However, due to the high support potential of surrogate-seeking for affected persons and the high relevance of the social support system in the context of mental health and illness [70], this seems worth investigating. The relevance of surrogate seeking, becomes particularly apparent in the context of a two-step-flow of communication, as surrogate seekers forward information to those persons who are unwilling (e.g., due to a lack of self-awareness as being at risk or affected), unable (e.g., due to their mental health status), or hampered (e.g., due to the persistent stigma) to search for and deal with such information themselves. Further, this seeking behavior is highly relevant for the dissemination and the overall outreach of mental health-related information, not only in case of an illness but also regarding prevention, social, or health policy issues.

In our study, we therefore want to take a more differentiated look at mental health information-seeking behaviors and ask the following:

RQ2: What sociodemographic and proximity factors are associated with the distinct types of information-seekers, i.e., people who seek information for themselves, people who seek information for others (surrogate-seekers), and people who seek for both themselves and others (self-and-surrogate-seekers)?

## Method

### Procedure

A face-to-face, paper-and-pencil survey was conducted among 3,042 German citizens aged 18 years and over in 2020 (response rate, 57%). Due to the coronavirus pandemic, participants could choose to fill out the survey themselves, which 15 percent of them did. The sample was drawn using a random sampling procedure with three stages: (1) sample points (electoral wards), (2) households, and (3) individuals within the target households. Target households within the sample points were determined according to the random route procedure, that is, a street was selected randomly as a starting point from which the interviewer followed a set route through the area. Target individuals within households were selected using random digits (cf. Kish grid, [71]). Informed consent was considered given when individuals agreed to complete the interview. The fieldwork was carried out by USUMA (Berlin, Germany), a company that is specialized in market and social research.

### Sample

Half of the total sample (*n* = 1,522) were asked to answer questions on mental health information-seeking and were thus considered in the analyses presented here. Participants, randomly assigned to this subsample, were 54 percent women and, on average, 49 years old (*SD* = 17.3 years). Of the respondents, 30 percent have no formal educational degree or completed only 8 or 9 years of schooling, while 42 percent have an intermediate (10 years of schooling) and 28 percent a high level of education (at least 12/13 years of schooling). Slightly more than half said they lived in a partnership; the others described themselves as single. Of the respondents, 82 percent do not have any migration background; either the remaining 18 percent moved to Germany themselves or their parents or grandparents migrated to Germany from another country. Among the respondents, 21 percent reported being in treatment due to a mental illness. The multistage sample design in our study ensures that each household has had the same probability of being included in the sample. Nonresponse nevertheless might have led to a bias in distribution compared to the population. A detailed sample description, also in comparison to population data, can be found in Table 1. The table shows that there are only minimal deviations in terms of key sociodemographic characteristics compared with the population as a whole.

**Table 1:** Characteristics of seekers, non-seekers, and the total sample in comparison to the German population

	**German Population**^a^	**Total** **(*n* = 1,522)** **100%**	**Seekers** **(*n* = 392)** **25.8%**	**Non-Seekers** **(*n* = 1,130)** **74.2%**	**Difference (*F* or** **chi-square statistics & Cramer’s *V*)**	**df**	***p*** **(two-tailed)**
Gender					χ2 = 12.382, *V* = .09	1	< .001
Male	48.9%	45.5%	37.8%	48.1%			
Female	51.1%	54.5%	62.2%	51.9%			
Age Group					χ2 = 3.936, *V* = .051	3	.268
18–25 years	10.4	11.4%	12.0%	11.2%			
26–45 years	29.9	32.5%	35.5%	31.5%			
46–60 years	27.3	28.0%	28.1%	28.0%			
> 60 years	32.4	28.1%	24.5%	29.3%			
∅ Age (in years) *(SD)*	-	48.6 (17.3)	47.2 (16.9)	49.1 (17.5)	*F* = 3.308	1	.069
Education					χ2 = 51.100, *V* = .184	2	< .001
Low (no or only 8/9 years of schooling)	34.5%	30.1%	32.4%	23.7%			
Intermediate (10 years of schooling)	30.7	41.5%	44.2%	33.9%			
High (12/13 years of schooling)	34.8	28.3%	23.5%	42.3%			
Relational status					χ2 = 1.346, *V* = .03	1	.246
Single/Divorced/Widowed	48%	43.7%	46.3%	42.9%			
Married/in partnership	52%	56.3%	53.7%	57.1%			
Migration background					χ2 = 4.915, *V* = .057	4	.296
No migration background	72.8%	81.7%	80.3%	82.1%			
Grandparents immigrated to GER	-	3.3%	4.1%	3.0%			
One parent immigrated to GER	-	3.8%	5.4%	3.3%			
Both parents immigrated to GER	-	3.8%	3.6%	3.8%			
Respondent him-/herself immigrated to GER	-	7.4%	6.6%	7.7%			
Proximity							
Self-affected by a mental illness	-	21.0%	50.5%	10.6%	χ2 = 280.201, *V* = .429	1	< .001
Person in close environment affected	-	29.4%	61.2%	18.3%	χ2 = 258.284, *V* = .412	1	< .001
Professional contact with affected persons	-	7.0%	20.4%	2.4%	χ2 = 144.574, *V* = .308	1	< .001
∅ Social distance *(SD)*	-	2.84 (0.91)	2.51 (0.79)	2.96 (0.92)	*F* = 73.580	1	< .001

^a^Data from the Federal Statistical Office of Germany [Statistisches Bundesamt (Destatis), 2020]; population data not for all sample characteristics available; data for educational attainment only available for persons ≥ 20 years

### Measures

Information seeking was assessed by asking whether participants have ever sought information on mental health or illness, regardless of where or for whom (Question: “Have you ever looked specifically for information about mental health or illness, anywhere or from anyone?”; Answer options: “Yes”, “No”, “I cannot remember”). If people have sought mental health information before, we subsequently asked whom people have sought this information for (Question: “And when you were researching this topic, who were you looking for the information for?”). The response options were “for myself,” “for someone else,” and “both for myself and someone else”. The original wording in German can be found in the supplementary file. Additionally, we asked for the participants’ sociodemographic characteristics (age, gender, education, relationship status, and migration background). We further assessed the respondents’ proximity to people with mental health issues, including personal mental health treatment experience and the desire for social distance from someone with depression, as described in a brief case vignette (nine items from Angermeyer & Matschinger [72], e.g., “To what extent would you accept someone like that as a work colleague? “; α = 0.92, *M* = 2.84 on a scale from 1 to 5, *SD* = 0.91, for all items see supplementary file). The example of depression was chosen because depression is the most prevalent and known mental disease [73].

### Statistical procedure

To address RQ1 and RQ2, we first performed simple statistical independence tests between the groups. Depending on the variable, either χ^2^ statistics and Cramer’s V*,* or ANOVA *F*-tests were used to test significant differences among the groups [74]. In a second step, we applied a multinomial, hierarchical logistic regression model, in which we compared self-seekers (*n* = 102), surrogate seekers (*n* = 144), as well as self- and surrogate-seekers (*n* = 139) each with non-seekers (*n* = 1,130) as the reference category. As independent variables, we included predisposing, sociodemographic factors (age, gender affiliation, education, relational status, and migration background) in the first step and proximity factors (mental health status, professional contact, affected people in environment, and social distance) in the second step. All analyses have been conducted using IBM’s SPSS® 28. The level of significance was set at 5 percent (i.e., *p* values lower than 0.05 were considered significant).

## Results

### Differences between seekers and non-seekers

Of the respondents, 26 percent indicated that they had already sought information about mental health or illness (*n* = 392). As shown in Table 1, seekers and non-seekers differ significantly in terms of gender and the highest level of schooling as well as contact with people affected and desire for social distance from mentally ill people. Although the effect size is rather small (Cramer’s *V* = 0.09, *p* < 0.001), women are significantly more likely than men to seek information on mental health or illness. The proportion of those who have already sought information on the topic of mental health or illness is linked to school education: Persons with 12 or 13 years of schooling inform themselves more frequently than persons with ten years of schooling, and persons with ten years of schooling do so more frequently than persons with only eight or nine years of schooling (Cramer’s *V* = 0.184, *p* < 0.001). The largest differences can be found for the proximity factors. Individuals directly or indirectly affected by a mental illness are significantly more likely to search for information on the topic than individuals without contact do. Above all, persons who are undergoing treatment for their own mental illness are most frequently among the seekers (Cramer’s *V* = 0.429, *p* < 0.001). Furthermore, people who have not yet sought information on the topic of mental health reported a significantly higher desire for social distance from persons with depression than people who already sought information (*F* [1, 1516] = 73.580, *p* < 0.001, η^2^ = 0.046). Differences between seekers and non-seekers were not significant in terms of age, relationship status, and migration background.

### Differences between distinct seeker types

Among all information seekers, 27 percent said they had sought information for themselves (*n* = 102), and 37 percent had sought information for someone else (*n* = 144), while the rest had done so for both themselves and others (*n* = 139). Table 2 compares the different types of seekers, namely self-seekers, surrogate seekers, and self- and-surrogate-seekers. Despite variations from a descriptive perspective, the types of seekers do not differ significantly in their sociodemographic characteristics, except education (Cramer’s *V* = 0.1721, *p* < 0.05). Nearly half of the people seeking for both themselves and others have a high education level, while self-seekers come from all educational groups equally. Additionally, differences emerge with respect to the proximity to affected persons. The proportion of people in treatment for a mental illness is highest among people who searched for information for themselves (Cramer’s *V* = 0.437, *p* < 0.001), while people with affected people in their environment are most likely to seek information for both themselves and others, as well as exclusively for others (Cramer’s *V* = 0.200, *p* < 0.001). Furthermore, people that are professionally or voluntarily involved in the care or treatment of people with mental illness, occur most frequently among those seeking for both themselves and others (Cramer’s *V* = 0.178, *p* < 0.01). Finally, people who search for information only for others differ significantly from people who search for themselves and others in terms of their desire for social distance: Surrogate-seekers (*M* = 2.65, *SD* = 0.77) have a significantly greater desire for social distance than self- and surrogate-seekers (*M* = 2.37, *SD* = 0.78), whose value is the lowest among all three seeker types (*F* [2,382] = 4.595, *p* < 0.05, η^2^ = 0.023).

**Table 2:** Characteristics of self-seekers, surrogate-seekers, and self- and surrogate-seekers

	**Self-** **Seekers** **(*n* = 102)**	**Surrogate-Seekers** **(*n* = 144)**	**Self- and-Surrogate-** **Seekers (*n* = 139)**	**Difference (*F* or** **chi-square statistics & Cramer’s *V*)**	**df**	***p*** **(two-tailed)**
Gender				χ2 = 2.319, *V* = .078	2	.314
Male	38.2%	32.9%	41.6%			
Female	61.8%	67.1%	58.4%			
Age Group				χ2 = 7.745. *V* = .100	6	.257
18–25 years	10.8%	10.4%	15.1%			
26–45 years	40.2%	29.2%	38.8%			
46–60 years	27.5%	30.6%	25.2%			
> 60 years	21.6%	29.9%	20.9%			
∅ Age (in years) *(SD)*	46.58 (15.89)	49.24 (17.42)	45.08 (16.96)	*F* = 2.207	2	.111
Education				χ2 = 11.260, *V* = .121	4	.024
Low (no formal educational degree or only 8/9 years of schooling)	31.4%	17.4%	25.2%			
Intermediate (10 years of schooling)	32.4%	41.7%	27.3%			
High (12/13 years of schooling)	36.3%	41.0%	47.5%			
Relational status				χ2 = 1.948, *V* = .072	2	.378
Single/Divorced/Widowed	52.5%	44.0%	44.9%			
Married/in partnership	47.5%	56.0%	55.1%			
Migration background				χ2 = 8.036, *V* = .102	8	.430
No migration background	82.4%	78.3%	80.6%			
Grandparents immigrated to GER	2.9%	4.9%	4.3%			
One parent immigrated to GER	3.9%	4.9%	6.5%			
Both parents immigrated to GER	6.9%	3.5%	1.4%			
Respondent him-/herself immigrated to GER	3.9%	8.4%	7.2%			
Proximity						
Self-affected by a mental illness	78.4%	24.3%	56.8%	χ2 = 73.586, *V* = .437	2	< .001
Person in close environment affected	47.1%	61.8%	71.9%	χ2 = 15.399, *V* = .200	2	< .001
Professional contact with affected persons	9.8%	20.1%	28.1%	χ2 = 12.136, *V* = .178	2	.002
∅ Social distance *(SD)*	2.52 (0.80)	2.65 (0.77)^a^	2.37 (0.78)^a^	*F* = 4.595	2	.011

Values marked with the same character ^a^differ significantly row-wise

The results of the logistic regression model examining the factors associated with belonging to the distinctive types of mental health information-seekers are depicted in Table 3. The strength of the association between each predictor variable and the outcome is expressed in the form of odds ratios (OR), indicating the expected change in the likelihood of observing the outcome (i.e., being a self-seeker, a surrogate-seeker, or a self-and-surrogate-seeker compared to being a non-seeker) when the respective predictor changes by one unit.

**Table 3:** Results of the multinominal logistic regression model (DV: Type of mental health information-seeking^a^)

	Determinant		Block 1			Block 2		
			B (SE)	OR	95% CI of OR	B (SE)	OR	95% CI of OR
**Non-Seekers vs** **Self-Seekers**	Socio-demographics	Age (in years)	-0.01 (0.01)	.994	[.982;1.006]	-0.01 (0.01)	.993	[.979;1.008]
		Gender (Ref: female)	-0.01 (0.01)	.663	[.435;1.011]	0.22 (0.25)	1.246	[.761;2.040]
		Level of education (Ref: low)						
		Intermediate	-0.27 (0.26)	.763	[.454;1.281]	-0.21 (0.3)	.808	[.449;1.456]
		High	0.44 (0.27)	1.558	[.920;2.636]	0.48 (0.31)	1.612	[.880;2.954]
		Relationship Status (Ref: single)	-0.34 (0.21)	.712	[.471;1.077]	-0.27 (0.24)	.761	[.477;1.214]
		Migration background	-0.04 (0.27)	.965	[.563;1.652]	-0.49 (0.31)	.613	[.332;1.131]
	Proximity	Self-affected***				3.4 (0.28)	29.914	[17.209;51.998]
		Person in the close environment affected				0.46 (0.25)	1.578	[.960;2.595]
		Professional contact with affected persons*				1.12 (0.44)	3.065	[1.288;7.295]
		Social Distance*				-0.37 (0.15)	.692	[.517;.926]
**Non-Seekers vs** **Surrogate Seekers**	Socio-demographics	Age (in years)	0.01 (0.01)	1.008	[.997;1.019]	0.01 (0.01)	1.008	[.996;1.020]
		Gender (Ref: female)*	0.01 (0.01)	.521	[.357;.761]	-0.46 (0.21)	.633	[.422;.949]
		Level of education (Ref: low)						
		Intermediate	0.58 (0.26)	1.793	[1.080;2.975]	0.48 (0.27)	1.616	[.949;2.753]
		High***	1.31 (0.27)	3.693	[2.188;6.233]	1.06 (0.29)	2.879	[1.647;5.032]
		Relationship Status (Ref: single)	-0.04 (0.19)	.963	[.669;1.385]	-0.1 (0.2)	.904	[.614;1.333]
		Migration background	0.33 (0.22)	1.385	[.894;2.144]	0.05 (0.24)	1.049	[.650;1.694]
	Proximity	Self-affected				0.38 (0.25)	1.455	[.891;2.376]
		Person in the close environment affected***				1.73 (0.21)	5.629	[3.770;8.405]
		Professional contact with affected persons***				1.72 (0.32)	5.586	[2.979;10.474]
		Social Distance				-0.17 (0.12)	.844	[.673;1.060]
**Non-Seekers vs** **Self-and Surrogate-Seekers**	Socio-demographics	Age (in years)	-0.01 (0.01)	.992	[.981;1.003]	-0.01 (0.01)	.991	[.978;1.004]
		Gender (Ref: female)	-0.01 (0.01)	.740	[.513;1.067]	0.27 (0.22)	1.306	[.840;2.029]
		Level of education (Ref: low)						
		Intermediate	-0.31 (0.25)	.732	[.448;1.194]	-0.44 (0.29)	.645	[.366;1.134]
		High*	0.83 (0.24)	2.287	[1.443;3.625]	0.58 (0.28)	1.787	[1.031;3.097]
		Relationship Status (Ref: single)	-0.02 (0.19)	.976	[.676;1.408]	-0.11 (0.22)	.899	[.587;1.377]
		Migration background	0.03 (0.24)	1.034	[.651;1.640]	-0.43 (0.28)	.652	[.375;1.134]
	Proximity	Self-affected ***				1.94 (0.24)	6.984	[4.391;11.108]
		Person in the close environment affected***				1.68 (0.23)	5.345	[3.396;8.411]
		Professional contact with affected persons***				2.23 (0.33)	9.335	[4.874;17.876]
		Social Distance***				-0.57 (0.14)	.563	[.429;.739]
Nagelkerke’s R^2^ *(Δ R*^*2*^*)*			.066			.429 (.363)		

Missing cases were deleted listwise, resulting in *n* = 1,479. *OR* Odds Ratio, *95% CI* Confidence Interval

^a^Coding of the dependent variable: 0 = non-seekers (Reference category); 1 = self-seekers; 2 = surrogate seeker; 3 = self- and surrogate-seekers

^*^p < .05, ** *p* < .01, ***p < .001 (two-tailed tests) – Significance level refer to the final level with two blocks

In the full model, the included variables explained 42.9 percent of outcome variance (i.e., whether the respondent was a self-seeker, surrogate-seeker, self-and-surrogate-seeker, compared to a non-seeker of mental health information). This high explanatory power originates primarily from the proximity factors added in the second step: They immensely increased the pseudo-R^2^ value (from 0.7 to 42.9 percent). The overall model fit is quite well, as indicated by the goodness-of-fit test comparing the full model with the empty model and yielding a significant result (1850.071, χ2 = 635.011, df = 30; *p* < 0.001).

Being in treatment for a mental illness increases the likelihood of self-seeking for mental health information the most (OR 29.914, 95 percent CI 17.209–51.998). Additionally, contact with affected persons in the work context is significantly related to self-seeking (OR 3.065, 95 percent CI 1.288–7.295), whereas a higher desire for social distance decreases the odds of looking for mental health-related information for oneself (OR 0.692, 95 percent CI 0.517–0.926). Sociodemographic characteristics are not associated with this seeking behavior.

This differs in the case of surrogate-seeking. Being a surrogate seeker, compared to being a non-seeker, is related with the seeker’s gender affiliation (OR 0.633, 95 percent CI 0.422–0.949), with women being more likely to seek information for someone else. Additionally, the respondents’ education level emerged as an important factor: People with high educational attainment (i.e., 12 or 13 years of schooling) are more likely to seek information for others (OR 2.879, 95 percent CI 1.647–5.032). There was, however, no association for people with an intermediate level of education compared to those with a low level of education. Being in treatment for a mental illness oneself was not related to surrogate-seeking, but proximity in terms of having someone affected in the close environment (OR 5.629, 95 percent CI 3.770–8.405) or due to professional contact (OR 5.58, 95 percent CI 2.979–10.474) was strongly associated with surrogate-seeking.

There is no evidence for a relationship between age, gender, relationship status, or migration background and being a self-*and-*surrogate-seeker, i.e., seeking information not only for oneself but also for others, compared to a non-seeker. However, a high education level was, in comparison to a low education level, associated with a nearly double-increase in the odds of being a self-and surrogate-seeker for mental health information (OR 21.787, 95 percent CI 1.031–3.097). Just as for surrogate-seeking, there was no relationship found for people with an intermediate level of education compared to those with a low level of education. Further, people who are themselves in treatment for a mental illness (OR 6.984, 95 percent CI 4.391–11.108) or who have people in their environment that are in treatment for a mental illness (OR 5.34, 95 percent CI 3.396–8.411) were significantly more likely to be self-and-surrogate-seekers. Especially, professional contact with affected people increased the odds by a factor of nearly ten (OR 9.335, 95 percent CI 4.874–17.876). Finally, desire for social distance emerged as an important factor with people having stigmatizing attitudes being less likely to be a self-and surrogate-seeker (OR 0.563, 95 percent CI 0.429–0.739).

## Discussion

The purpose of our study was to closely examine information-seeking in the context of mental health. We aimed to identify distinctive patterns of sociodemographic and proximity-related factors that help to explain differences between non-seekers, self-seekers, and surrogate-seekers. It is a merit of this paper to include the group of surrogate seekers as, in the context of mental health stigma, there are especially high barriers to help-seeking, which raises the crucial role of social support by information acquisition on behalf of those who suffer from mental health problems [75].

Our analysis revealed that around a quarter of the German adults in our sample have already searched for information on mental health. Comparing this finding with a recent population survey on health information-seeking in general, which is conducted by around 75 percent of the overall German population [45], we can conclude that searching information about mental health has a notable share in health-related information-seeking behaviors. The total share of mental health information seekers found in our study is consistent with findings from the US as, in a 2009 study, about 20 percent of the population reported having searched for mental health-related information at some point [22].

Among those people in our sample who searched for mental health information, we found more than 70 percent of surrogate seekers. This proportion appears to be slightly higher for the specific topic of mental health than for surrogate-seeking of health information in general, which in previous studies accounted for about 60 percent in European countries [66] or two thirds in the US [1]. This can be explained by the crucial role of mental health stigma: Research on the impact of stigma on help-seeking has shown that the individuals’ own stigmatizing attitudes constitute a barrier to help-seeking [12], which is consistent with our finding, that a greater desire for social distance is negatively associated with information-seeking for oneself. Thus, self-distancing takes place both on an attitudinal level and an information behavioral level, which may result from a lack of interest or an active avoidance strategy to not scrutinize one’s own established opinion (cognitive consonance, see [76, 77]).

Our results on the factors associated with mental health information seeking are mostly consistent with previous research. Particularly, we found that as for general health information behavior [13, 19, 53, 78] or cancer-related information behavior [56, 79], gender and education are significantly but weakly related to mental health information-seeking, while health status, or rather experience with mental health treatment, plays a vital role in mental health information-seeking. This also agrees with the few prior studies on mental health information-seeking [14, 15, 21, 60]. As men are significantly less likely to seek treatment if they become mentally ill [80] and at the same time are significantly more prone to fatal suicide [81], their lagging behind in information-seeking appears problematic. However, it should be considered that men may eventually just use different terms to describe mental health and illness beyond the nomenclature of mainstream psychiatry (e.g., stress, emotional discomfort), and that the found differences are maybe due to a different perception of the terms “mental health and illness”. Further, men may use different ways to deal with mental health problems or emotional distress than seeking information (e.g., coaching, [82]).

Likewise, the educational gap is critical, as lower socioeconomic status is associated with a higher prevalence of mental illness despite more difficult access to the mental healthcare system [83, 84]. The reasons for the differences could be distinct motives for seeking information, as well as different risk perceptions [78]. Furthermore, the access to available information and its content and design may not adequately meet the needs of men and people with lower education.

As with the findings of Lannin and colleagues [60], we found that the individuals’ personal contact with people affected by a mental disorder turns out to be a key factor for mental health information-seeking. The share of people with any form of proximity to mental health disorders is significantly higher among mental health information-seekers than -non-seekers. Furthermore, it is highly plausible that self-seeking is closely linked to being affected and treated oneself, while having someone affected in the nearby environment is more prevalent among those who search for information on mental health for others.

However, differences emerge when looking at the results of the multinomial logistic regression. Whether someone searches for mental health information for oneself or others is, compared to non-seeking, only related to people’s own health status, professional contact with the people affected, and their attitude towards mentally ill people. Sociodemographic factors do not seem to play a role at all. Self-seekers can thus be described as strongly self-involved.

Surrogate seekers fit the image of people who care about others and therefore also seek information for others. They, for the most part, are female, have an intermediate education, and have the highest average age of all seeker types. Their interest in the topic seems to arise primarily from the affected people in their environment, be it privately (e.g., in a mother’s role) or professionally (e.g., as a caregiver). These characteristics match with prior research on surrogate seekers in general [1, 66] and show that relationship closeness, especially, as Reifegerste and Bachl [69] noted, plays an important role in surrogate-seeking.

People looking for information both for themselves *and* others are highly involved. They are characterized by a high level of education and by a strong closeness to the subject such as being in treatment themselves and/or having persons in mental health treatment in the private or professional environment. They also have the lowest desire for social distance among the three seeking types and can therefore possibly be characterized as ‘highly involved mental health advocates.’

Overall, our results point to the fact that individuals’ social and attitudinal proximity to the topic and to the persons affected is much more associated with mental health information-seeking than sociodemographic and socioeconomic factors. This subordination of sociodemographic in favor of individual health-related cognitive and sociopsychological factors aligns with theoretical assumptions and prior research on health information-seeking behaviors. Studies considering sociodemographic, health-related proximity factors, and/or motivational or cognitive factors confirm that the relative impact of sociodemographic determinants is comparatively low [13, 19, 85].

Thus, we conclude that communication efforts in mental health promotion should focus less on sociodemographic or socioeconomic factors as the usual target group segmentation strategies but rather address target groups with a view to how they relate to the topic. We recommend addressing persons as being affected and/or as supporters or caregivers to those affected. This holds the potential for people to feel personally addressed, identify with their role, and be open to corresponding informational support.

### Limitations

The interpretation of our results requires consideration of some key limitations. First, we deal with cross-sectional data that do not allow for a conclusive determination of causality. For example, it is reasonable to assume not only that desire for social distance is a (negative) predictor of seeking mental health information, but conversely, that lack of contact with information produces more stigmatizing attitudes. On the other hand, our data are based on self-reports, so biases regarding social desirability cannot be ignored entirely.

In addition, despite being fully within the expected range for face-to-face population based random samples, the response rate can be considered low and we cannot rule out a self-selection bias. Therefore, our results can only be generalized to a limited extent.

As a limitation, it must also be said that we assessed mental health information-seeking very broadly. We did not ask about specific channels or topics. Moreover, the answers refer to the search for information on *both* mental health *and* illness, i.e., a very general topic. Building on the findings presented here, further research should be more nuanced and examine channel- as well as topic-specific differences. Finally, the list of included determinants is far from complete. For instance, the respondents’ income or their (digital) health literacy—both well known to be associated with health information seeking (e.g., [86, 87])—were not assessed in this survey. Future research should therefore investigate more influencing factors beyond those analyzed in this study.

## Conclusions

We were able to show that mental health information seekers differ significantly from one another when we differentiate whom they are searching information for. We claim to consider surrogate seekers as an important and distinctive target group for mental health information provision. This should also be taken into account in future studies and communication efforts. Depending on whether one wants to promote surrogate- or self- seeking, different target groups and determinants should be addressed. Future research should also delve deeper into what channel- and content-specific differences and predictors exist and, most importantly, identify additional barriers and obstacles to searching for mental health-related information. In summary, our findings provide valuable inputs for strategic mental health communication and targeting. They emphasize the importance of providing access to mental health information and to thus potentially increase the rate of those who seek help quickly and purposefully when they fall ill.

## Supplementary Information

**Additional file 1.**

**Additional file 2.**

## Data Availability

All data analysed during this study are included in this published article and its supplementary information files.
