# Supplementary material for: Self- and surrogate-seeking of information about mental health and illness in Germany
Source: BMC Public Health. 2023 Jan 10;23:65. doi: 10.1186/s12889-023-14998-0 (PMC9830618; doi:10.1186/s12889-023-14998-0)
Supplement: Supplementary file 2 — Additional file 2. [file 12889_2023_14998_MOESM2_ESM.docx]

**Supplementary File for**

**“Self- and Surrogate-Seeking of Information about Mental Health and Illness in Germany: A Population Survey”**

Table A1

Assessment of Information Seeking: Exact Wording in English and German

|  | **Question** | **Answer options** |
| --- | --- | --- |
| GER | Haben Sie sich auch schon einmal gezielt über das Thema psychische Gesundheit oder Krankheit informiert, egal wo oder bei wem? | Ja \| Nein \| Kann ich mich nicht dran erinnern |
| EN | Have you ever looked specifically for information about mental health or illness, anywhere or from anyone? | Yes \| No \| I can not remember |
| GER | Und als Sie sich zu diesem Thema informiert haben, für wen haben Sie die Informationen gesucht? | Für mich selbst \| Für jemand anderen \| Sowohl für mich als auch für jemand anderen |
| EN | And when you were researching this topic, who were you looking for the information for? | For myself \| For someone else \| Both for myself and someone else |

Table A2

Social Distance: Items, Results, and Reliability

| **Item** | *M* (*SD*) |
| --- | --- |
| If you had a room to rent, to what extent would you accept someone with such a problem as a subtenant? *(GER: Wenn Sie ein Zimmer zu vermieten hätten, inwieweit würden Sie jemanden mit einem solchen Problem als Untermieter nehmen?)* | 3.07 (1.2) |
| To what extent would you accept someone like that as a work colleague? *(GER: Inwieweit würden Sie so jemanden als Arbeitskollegen akzeptieren?)* | 2.5 (1.12) |
| To what extent would you accept such a person as a neighbor? *(GER: Inwieweit wäre Ihnen so jemand als Nachbar recht?)* | 2.4 (1.12) |
| To what extent would you entrust your children to someone like that for a few hours for supervision? *(GER: Inwieweit würden Sie so jemandem Ihre Kinder für einige Stunden zur Aufsicht anvertrauen?)* | 3.74 (1.15) |
| To what extent would you accept such a person marrying into your family? *(GER: Inwieweit wären Sie damit einverstanden, dass so jemand in Ihre Familie einheiratet?)* | 3.15 (1.16) |
| To what extent would you introduce a friend of yours to such a person? *(GER: Inwieweit würden Sie eine Freundin von Ihnen mit so jemandem bekannt machen?)* | 2.97 (1.19) |
| If one of your friends had a job to fill, to what extent would you recommend such a person to them? *(GER: Wenn einer Ihrer Bekannten eine Arbeitsstelle zu besetzen hätte, inwieweit würden Sie ihm dann so jemanden empfehlen?)* | 3.45 (1.13) |
| To what extent would you be willing to talk to such a person? *(GER: Inwieweit wären Sie bereit, sich mit so einer Person zu unterhalten?)* | 1.9 (1.06) |
| To what extent would you invite someone like that to your home? *(GER: Inwieweit würden Sie so jemanden zu sich nach Hause einladen?)* | 2.41 (1.23) |
| Scale Mean (SD) | 2.84 (0.91) |
| Cronbach’s Alpha | .92 |

Note: *N* = 1,526. All items were assessed on a five-point Likert scale from 1 (full agreement) to 5 (no agreement) via the question: “Finally, I would be interested to know how you relate to someone with such a problem (beforehand, a vignette describing a person with depression was presented). I’m going to read you a few questions about this. Each time, please tell me what your attitude is using the scale on the list.”
